# Treg cell plasticity as a driver of inflammation in spondyloarthritis and psoriasis

**DOI:** 10.3389/fimmu.2025.1621396

**Published:** 2025-07-24

**Authors:** Ingrid Itzayanna Ortega-Mejia, Nayeli Romero-López, Julio César Casasola-Vargas, Rubén Burgos-Vargas, María Lilia Domínguez-López, José Pablo Romero-López

**Affiliations:** ^1^ Laboratorio de Patogénesis Molecular, Edificio A4, Carrera de Médico Cirujano, Facultad de Estudios Superiores Iztacala, Universidad Nacional Autónoma de México (UNAM), Tlalnepantla de Baz, Estado de Mexico, Mexico; ^2^ Laboratorio de Inmunoquímica 1, Posgrado en Ciencias Quimicobiológicas, Escuela Nacional de Ciencias Biológicas, Instituto Politécnico Nacional, Ciudad de Mexico, Mexico; ^3^ Department of Rheumatology Hospital General Dr. Eduardo Liceaga, Ciudad de Mexico, Mexico

**Keywords:** Treg cells, Treg plasticity, IL-17, spondyloarthritis, psoriasis

## Abstract

Regulatory T cells (Tregs) are critical for maintaining immune tolerance by suppressing effector T cell responses. However, in chronic inflammatory diseases such as spondyloarthritis (SpA) and psoriasis (PsO), Tregs can lose their stability and acquire pro-inflammatory characteristics, a phenomenon known as Treg plasticity. Under inflammatory conditions, Tregs may downregulate FoxP3, upregulate RORγt, and produce cytokines such as IL-17 and IFN-γ, thus losing their suppressive function and contributing to disease progression. In SpA, altered numbers and impaired Treg function have been identified in peripheral blood and synovial fluid. Specific subsets, such as CD161^+^ Tregs with Th17-like features, suggest that inflammatory cytokines and signals like STAT3 activation and ICOS engagement promote pathogenic reprogramming. Genetic factors, including HLA-B27, may further predispose Tregs to instability. Single-cell transcriptomic analyses have provided evidence of shared TCR repertoires between Tregs and effector T cells, reinforcing the concept of lineage plasticity. Similarly, in PsO, skin-resident Tregs exposed to IL-23, IL-6, and IL-21 can acquire a Th17-like phenotype, producing IL-17A and exacerbating local inflammation. Environmental factors such as hypoxia also contribute to destabilizing Treg identity. The persistence of pathogenic Tregs, even following therapy blockade of IL-17 or IL-23, highlights the challenge of achieving long-term disease remission.

## 1 Introduction

Regulatory T lymphocytes, known as Treg, were first phenotyped in 1995 as a specific subpopulation of immune cells that could suppress effector T cells (1). These cells are characterized by high expression of the CD25 molecule, which corresponds to the alpha chain of the interleukin-2 receptor (IL-2R) (2). Their main function is to maintain immune self-tolerance, preventing the immune system from mistakenly attacking the body’s own tissues. To achieve this, Treg lymphocytes modulate effector immune mechanisms. Under normal conditions, they comprise approximately 5-10% of total CD4+ T lymphocytes (3).

One of the most important characteristics in the development and function of Treg lymphocytes is the high expression of the FoxP3 transcription factor (4), which acts as a “master regulator” that guides the differentiation of these cells toward the regulatory lineage and establishes their immunosuppressive identity (5). Additionally, FoxP3 stability is essential for maintaining Treg suppressive function; therefore, it plays a key role in sustaining immune tolerance (6).

Tregs expressing FoxP3 can be subdivided into two major subsets according to their origin. Thymic-derived Tregs (tTregs) develop in the thymus, primarily during the process of negative selection of autoreactive T lymphocytes (7). In contrast, peripheral Tregs (pTregs) arise from naive CD4^+^ T cells in peripheral tissues, where their differentiation is induced by cytokines such as transforming growth factor-beta (TGF-β) and interleukin-2 (IL-2) (8, 9).

Regardless of their origin, it has been proven that Treg cells play an immunoregulatory role through several common mechanisms (10). These include direct cell-to-cell interaction, local depletion of IL-2 due to high CD25 expression, and the expression of co-inhibitory molecules such as CTLA-4 and PD-1 (11, 12). Additionally, Tregs can deplete tryptophan, an essential substance for the function of other immune cells, and release suppressive cytokines such as IL-10, TGF-β, and IL-35, thereby limiting CD4+ T cell activation (13).

In addition to their immunoregulatory function, Treg cells can induce direct cytotoxic destruction of other cells (11). This process is mediated by cell-to-cell contact, involving inhibitory receptor-ligand pairs such as PD-1/PD-L1 and CTLA-4/CD80/CD86, and the direct production of granzymes, triggering apoptosis in target cells (14).

Treg lymphocytes require several activation stimuli to exert their regulatory function. One of the main ones is the recognition of their specific antigen, which can be presented by dendritic cells or other antigen-presenting cells (APCs) (15). Additionally, Tregs respond to cytokine signals such as interleukin-2 (IL-2) or TGF-β (16). Although Treg activation is typically antigen-specific, several antigen-independent pathways have also been identified (17).

The maintenance of immune tolerance theoretically depends on the functional stability of regulatory T (Treg) cells. However, Treg cells can exhibit a remarkable phenotypic plasticity, which is the ability to adopt the functional and phenotypic characteristics of other CD4^+^ T cell subtypes (18). This plasticity is primarily manifested through the co-expression of FoxP3 and other lineage-defining transcription factors like RORγt (19). Although Treg plasticity has been previously recognized, recent advances in single-cell transcriptomics and high-dimensional profiling have significantly expanded our understanding of this phenomenon, especially in chronic inflammation.

Multiple recent developments underscore the need for a comprehensive review of this topic. First, single-cell RNA sequencing studies have revealed a previously unappreciated level of heterogeneity among Treg subsets (20). While further epigenetic profiling could provide deeper insight into the mechanisms driving this diversity, the most up-to-date studies have relied primarily on single-cell transcriptomics, TCR sequencing, and functional assays. Techniques such as ATAC-seq, which could elucidate chromatin accessibility and regulatory element dynamics, remain underexplored in this context. Second, accumulating evidence indicates that Tregs (21) not only lose their suppressive function under inflammatory conditions but can also acquire pro-inflammatory features, thereby actively contributing to disease chronicity. Finally, immunotherapeutic strategies—particularly IL-17 and IL-23 blockade—have been shown to modulate Treg phenotype and function, prompting critical questions about their role in shaping therapeutic responses and long-term immune regulation (22).

An early example of regulatory T cell plasticity was observed in murine models, where a subset of adaptive regulatory T cells emerged during Th1-type immune responses. These cells, derived from CD4^+^CD25^-^ T lymphocytes, co-expressed the transcription factors FoxP3 and T-bet, and were capable of producing interleukin-10 (IL-10) as well as interferon-gamma (IFN-γ). This phenotype suggests a regulatory role adapted to inflammatory environments characterized by Th1 polarization (23).

A particularly relevant study was conducted in a *Toxoplasma gondii* infection model, showing that T-bet expression in Tregs was essential to prevent lethal immunopathology (24). During this infection, the loss of IL-2 promoted the conversion of Tregs toward an IFN–γ–producing phenotype, illustrating their adaptation to a Th-1-driven inflammation environment. This functional shift suggests that, despite acquiring effector-like features, these adapted Tregs can maintain their suppressive function and prevent severe immune-mediated damage (24).

An additional key feature of Treg plasticity is the stability of FoxP3 expression Although it has traditionally been referred to as a reliable marker of Treg identity and function, recent research has revealed that its expression can be unstable under certain conditions (4). There are CD4^+^ T cell populations that, while expressing FoxP3, do so transiently or unstably, raising questions about their true commitment to a regulatory phenotype. This variability has generated some controversy in the field, as some studies argue that Tregs remain stable as long as they express FoxP3. In contrast, others suggest that they may lose this identity under specific conditions, thereby broadening the scope of their functional plasticity (25). The identity of regulatory T cells (Tregs) is often defined by their immunosuppressive function. Consequently, if both FoxP3^+^ and FoxP3^-^ T cells are capable of suppressing effector T cell responses, the question of whether they should be classified as Tregs remains unresolved. Conversely, it is also debatable whether CD25^+^FoxP3^+^ T cells that have lost their suppressive capacity should continue to be considered *bona fide* Tregs. The confusion between the transient expression of *Foxp3* in activated T cells and true Treg identity contributes to the ongoing debate surrounding their plasticity (26).

Phenotypic stability of Treg largely depends on epigenetic regulation, particularly DNA demethylation within the TSDR region of the Foxp3 gene (27). While thymus-derived Tregs exhibit complete demethylation and stable Foxp3 expression, those induced *in vitro* often show partial demethylation and transient expression (28). Agents such as azacitidine can induce Foxp3 expression without the need for TGF-β, provided effective demethylation is achieved (29). *In vivo* strategies have also been shown to promote this stability. Based on these findings, future research could explore mechanisms to enhance stable TSDR demethylation in induced Tregs, investigate epigenetic therapeutic combinations in autoimmune diseases, and assess whether these mechanisms can be specifically targeted in various inflammatory contexts or tumors (30).

Beyond epigenetic control, metabolic reprogramming plays a central role in regulating Treg cell plasticity, stability, and function. Key biochemical pathways—including molecules such as mTOR (mechanistic target of rapamycin) and HIF-1α (hypoxia-inducible factor-1 alpha)—act as metabolic sensors that determine how Treg cells adapt to environmental conditions, influencing their ability to maintain suppressive activity or differentiate toward alternative T cell phenotypes. In Treg cells, mTOR activity promotes glycolysis and inhibits fatty acid metabolism (31). Its sustained activation destabilizes the Treg phenotype by downregulating FoxP3 expression. Accordingly, rapamycin has been used *in vitro* to stabilize Treg function and maintain regulatory identity (32, 33). On the other hand, under hypoxic conditions, the expression of HIF-1α in Tregs promotes glycolysis and facilitates differentiation into inflammatory phenotypes such as Th17 cells. This interaction between metabolic pathways and cell differentiation illustrates the high sensitivity of Tregs to their microenvironment (34).

For instance, under type 1 inflammatory conditions rich in IL-12 and IFN-γ, Tregs can upregulate the transcription factor T-bet and express the chemokine receptor CXCR3, facilitating their migration to Th1-dominated sites and allowing them to regulate local inflammation without losing suppressive capacity (35). This phenotype, known as Th1-like Tregs, has been shown to retain FoxP3 expression and mediate control of excessive Th1 responses through the secretion of IL-10 and the expression of cytotoxic molecules such as granzyme B (36). Similarly, Th2-like Tregs arise in environments rich in IL-4 and are characterized by GATA3 expression, which supports FoxP3 stability and promotes Treg homeostasis, especially at barrier surfaces like the skin and lungs (37).

**Figure 1:**
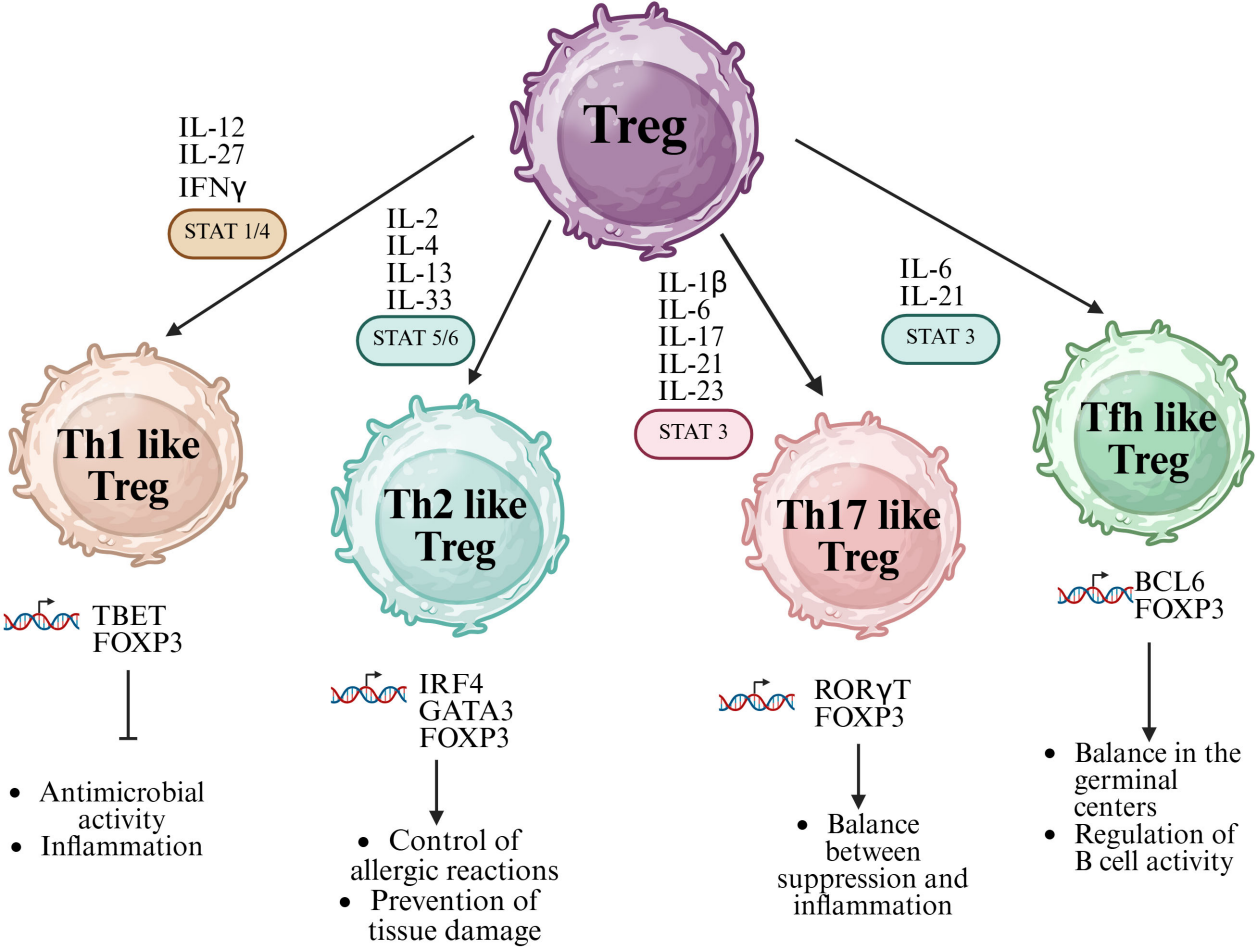
Functional specialization and plasticity of regulatory T cells (Tregs). FoxP3⁺ Tregs can differentiate into specialized subsets that mirror effector T helper (Th) lineages, acquiring phenotypic and functional characteristics of Th1, Th2, Th17, and T follicular helper (Tfh) cells. This plasticity is driven by cytokine-mediated activation of specific STAT signaling pathways: STAT1/4 for Th1-like Tregs, STAT5/6 for Th2-like Tregs, and STAT3 for both Th17-like and Tfh-like Tregs. Each subset expresses lineage-specific transcription factors (e.g., T-bet, GATA3, RORγt, Bcl6) and secretes corresponding cytokines, enabling context-specific regulation of immune responses. Created in BioRender. Romero, P. (2025) https://BioRender.com/p29m2dc.

In Th17-polarizing environments—often associated with the presence of IL-6, IL-1β, and TGF-β—Tregs may acquire features of Th17 cells through STAT3 signaling and upregulation of RORγt, giving rise to Th17-like Tregs (38). These cells have been reported to co-express FoxP3 and RORγt and may either retain suppressive function or contribute to pathology depending on the inflammatory context (39). Likewise, in germinal center reactions, a subset of follicular regulatory T cells (Tfr) expressing both FoxP3 and Bcl-6 emerges, driven by IL-21 and ICOS signaling. These Tfh-like Tregs limit the expansion of follicular helper T cells and prevent autoantibody production, representing a critical regulatory axis in humoral immunity (40) (**Figure 1**).

Similar to FoxP3, members of the Ikaros Zinc Finger (*IkZF*) transcription factor family have well-documented roles in immune cell development. Ikaros, the first transcription factor described in this family, was reported as essential for the development of lymphoid cells (41). Over the following decades, four additional proteins with high structural homology to Ikaros were identified, and these now comprise the IkZF transcription factor family: Ikaros (encoded by the *Ikzf1* gene), Helios (*Ikzf2*), Aiolos (*Ikzf3*), Eos (*Ikzf4*), and Pegasus (*Ikzf5*) (41).

The members of the IkZF family contain both an N-terminal DNA-binding zinc finger domain and a C-terminal protein-protein interaction domain (42). All members of this family are expressed within the hematopoietic compartment. While Ikaros is broadly expressed across all hematopoietic cells, Aiolos is predominantly found in cells committed to the lymphoid lineage and is highly expressed in mature B cells, whereas Helios expression is largely restricted to the T cell lineage^32^.

This structural diversity provides these factors with functional versatility, as *IkZF* members can regulate gene expression both positively and negatively by recognizing target DNA sequences and forming transcriptional complexes with other proteins. Mechanistically, *IkZF* factors have been shown to regulate gene expression through (i) chromatin remodeling by associating with chromatin remodeling complexes such as the nucleosome remodeling and deacetylase (NuRD) complex, (ii) initiation of transcription at the RNA Pol II level, and (iii) inducing chromosomal conformational changes by mediating interactions between distal cis-regulatory regions (43).

In recent years, the expression of the transcription factor Helios in regulatory T cells (Tregs) has been a subject of extensive investigation. It was initially proposed that Helios could serve as a marker to distinguish between thymic-derived Tregs (tTregs) and peripherally induced Tregs (pTregs), given its higher expression in tTreg cells (44). However, subsequent studies have challenged this notion, demonstrating that Helios can also be expressed in a subset of pTregs. In particular, Shevach and colleagues showed that both tTregs and pTregs can express Helios, and that Helios^+^ and Helios^-^ Treg subpopulations differ in their TCR repertoire, stability, and functional properties (43, 45). Complementarily, Helios is highly expressed in Treg cells, and it has been suggested that it plays a crucial role in their suppressive function (46). Furthermore, Helios enhances the transactivation of *Foxp3* by binding directly to its promoter, and Treg cells lacking Helios tend to lose their immunosuppressive function, resulting in the downregulation of *Foxp3* expression (46). Due to its close relationship with FoxP3, Helios plays a crucial role in maintaining the stability and functionality of Treg cells. However, it remains unclear whether the members of the Ikfz family can influence the conversion of the Treg phenotype into other lineages.

Despite this controversy, the functional role of Helios in Tregs has gained relevance, particularly due to its involvement in maintaining the phenotypic and functional stability of these cells. Helios participates in the epigenetic silencing of genes such as IL-2, thereby restricting Treg cell proliferation, supporting their suppressive capacity (47). Helios deficiency has been associated with a significant loss of this function, especially in contexts where Tregs are required to control the expansion of pathogenic T cells or mediate follicular regulatory T cell functions (48). Additionally, Helios-positive Tregs have been shown to exhibit a more activated phenotype, higher suppressive capacity, and greater stability in Foxp3 expression, particularly under conditions of immunological stress or lymphopenia (49). Taken together, these findings suggest that while Helios may not be a reliable marker for determining Treg origin, it is an essential factor in preserving their identity and limiting their plasticity, thus positioning Helios as a key regulator of the functional integrity of these cells.

Within Treg plasticity, two main phenomena have been identified: the emergence of ex-Treg cells and the development of “fragile” Treg cells. Ex-Tregs are cells that have completely lost expression of the transcription factor Foxp3, which is essential for maintaining Treg identity and suppressive function. Once Foxp3 is lost, these cells cease to act as immune regulators and may even acquire pro-inflammatory properties, contributing to processes such as autoimmunity or tumor rejection. In contrast, fragile Tregs retain Foxp3 expression but display reduced suppressive capacity and begin to express inflammatory genes, representing an intermediate form of dysfunction. Although the loss of Foxp3 is considered the main criterion for defining ex-Tregs, there is currently no universal consensus regarding additional markers or functional tests for their precise identification. This field remains under active investigation, and researchers continue to refine the definitions as knowledge of Treg fate and behavior deepens (50). This plasticity, while posing a challenge to our understanding of the immune system, also offers considerable therapeutic potential: on one hand, destabilizing Tregs may enhance anti-tumor immunity; on the other, stabilizing them could be key to reducing chronic inflammation in autoimmune diseases. Thus, understanding the mechanisms that regulate this transformation is essential for developing new clinical strategies.

In human studies, ex-Tregs have been identified as a distinct subset of CD4^+^ T cells with cytotoxic capabilities. Research by Freuchet et al. characterized these cells as CD3^+^CD4^+^CD16^+^CD56^+^ lymphocytes that express cytotoxic molecules such as granzyme B and perforin. These ex-Tregs lack suppressive function and instead exhibit properties similar to natural killer (NK) cells, including the ability to degranulate and eliminate target cells (51). The study also found that ex-Tregs are clonally related to conventional Tregs, suggesting a lineage relationship and highlighting the dynamic nature of Treg plasticity in human immune responses. In murine models, Treg instability and their conversion into ex-Tregs have also been associated with the loss of Foxp3 expression, leading to the acquisition of pro-inflammatory functions. In experimental autoimmune encephalomyelitis (EAE), a model for multiple sclerosis, Tregs that lose Foxp3 expression can contribute to disease progression by producing inflammatory cytokines such as IFN-γ and IL-17 (52). Similarly, in models of atherosclerosis, the conversion of Tregs into ex-Tregs is accelerated, exacerbating vascular inflammation (53).

Given the dynamic regulatory capacity of Tregs and their ability to adapt phenotypically and functionally to local inflammatory environments, understanding how this plasticity manifests in specific immune-mediated diseases has become a central research focus. This Treg plasticity has been documented in a range of autoimmune and inflammatory conditions, including multiple sclerosis (MS) (54), rheumatoid arthritis (RA) (55), giant cell arteritis (56), idiopathic orbital inflammation (57), and primary Sjögren’s syndrome (58). In these diseases, Tregs can acquire the ability to produce IL-17A and/or IFN-γ—often co-expressing transcription factors such as RORγt and displaying surface markers like CD161 and ICOS—particularly in inflamed tissues. This phenotypic shift is driven by pro-inflammatory cytokines (e.g., IL-1β, IL-21, IL-23), Toll-like receptor signaling, and interactions with antigen-presenting cells. Importantly, these plastic Tregs frequently exhibit reduced suppressive function and have been associated with heightened disease activity and immune dysregulation. Emerging evidence also suggests that this conversion is regulated by epigenetic mechanisms, further underscoring its relevance across diseases.

In chronic inflammatory disorders such as spondyloarthritis (SpA) and psoriasis (PsO), where dysregulated immune responses and tissue-specific inflammation are hallmarks of disease, accumulating evidence suggests that altered Treg function and stability contribute to disease pathogenesis. Therefore, elucidating the phenotypic shifts, transcriptional profiles, and functional outcomes of Treg plasticity in these contexts is essential to understand their role as regulators and potential drivers of inflammation.

## 2 Spondyloarthritis and Treg cell plasticity

Spondyloarthritis (SpA) is a group of chronic inflammatory disorders that primarily affect the axial skeleton, peripheral joints, and entheses. It includes clinically heterogeneous entities such as axial radiographic or non-radiographic SpA (ax-rSpA, ax-nrSpA), peripheral SpA (pSpA), psoriatic arthritis (PsA), reactive arthritis (ReA), and undifferentiated SpA (uSpA) (59). Among these, ax-rSpA (previously termed ankylosing spondylitis (AS)) is the best characterized and most studied. The disease predominantly affects young males and is strongly associated with the HLA-B27 allele. However, its presence alone is insufficient to cause disease, indicating that environmental triggers and other genetic factors are also involved in pathogenesis (60).

Clinically, SpA is classified into axial and peripheral forms, depending on whether the predominant manifestations involve the axial skeleton or peripheral joints and entheses (61). Its immunopathology is driven by complex interactions between the innate and adaptive immune systems, involving dysregulation of T cells and innate lymphocytes and overproduction of cytokines such as TNF-α, IL-17, and IL-22, which promote chronic inflammation and tissue remodeling (62).

Advancements in imaging and molecular characterization have led to the development of targeted therapies that improve disease control. TNF inhibitors remain a cornerstone in SpA treatment (63). Still, biologics directed against IL-17A, such as secukinumab and ixekizumab, have shown comparable or superior efficacy in certain clinical subsets, particularly in patients with axial involvement or psoriatic features (64, 65). Furthermore, small-molecule inhibitors targeting the JAK-STAT pathway, including tofacitinib and upadacitinib, are being incorporated into treatment algorithms due to their ability to interfere with multiple inflammatory cascades simultaneously (66).

Despite these therapeutic advances, disease remission remains elusive in many patients. Considering that Treg cell-directed immunotherapies are novel and functional strategies in other immune-mediated diseases, they are an emerging area of interest in this field. In SpA, alterations in the number, phenotype, and function of Tregs have been reported. A recent meta-analysis identified reduced circulating CD4^+^CD25^+^FoxP3^+^ Tregs in patients with active ankylosing spondylitis, suggesting a disrupted balance between regulatory and effector mechanisms (67). Similarly, McTaggart et al. found that Tregs from the synovial fluid of patients with psoriatic arthritis displayed diminished suppressive activity on autologous T cells (68).

Recent transcriptomic analyses have further characterized this dysfunction. Pouw et al. performed single-cell RNA sequencing of immune cells from inflamed joints of patients with ankylosing spondylitis and psoriatic arthritis, revealing that synovial Tregs exhibited a distinct transcriptional signature associated with reduced expression of co-inhibitory molecules and impaired regulation of myeloid cell activation (21). These findings indicate that Tregs in SpA are reduced in number and may also become functionally reprogrammed by the inflammatory environment.

Adding to this complexity, Simone et al. identified a subset of Tregs expressing CD161 (encoded by KLRB1) in blood and synovial fluid from patients with SpA. These CD161^+^ Tregs exhibit features overlapping with Th17 cells, including the capacity to produce IL-17 (69). Interestingly, these cells showed low expression of Helios, suggesting either peripheral induction or destabilization in the inflammatory milieu. The diverse Treg cell subsets discovered in this study include a cytotoxic CD8^+^ regulatory subset and a Th17-like subset characterized by IL-10 and LAG-3 expression.

These Th17-like Tregs may play a role in modulating IL-17-driven inflammation, although their exact function in disease pathogenesis requires further investigation. In support of this, Duurland et al. demonstrated that CD161^+^ Tregs and CD161^+^ conventional T cells (Tconv) share a transcriptional and functional profile despite a limited overlap in their TCRβ repertoires (70).The presence of these cells in inflamed tissues and their effector-like characteristics underscores their potential contribution to chronic inflammation when regulatory mechanisms fail.

Various factors influence the stability of Tregs in SpA. Studies have shown that Tregs from axSpA patients exhibit decreased expression of EZH2 and phosphorylated STAT5 (pSTAT5), both critical for maintaining Treg stability and suppressive function. Interestingly, treatment with TNF inhibitors has been associated with the restoration of pSTAT5^+^ Treg frequencies, suggesting that therapeutic modulation can partially reverse Treg instability (71).

Using scRNA-seq combined with TCR-seq, a study demonstrated that patients with ankylosing spondylitis exhibit a high proportion of dual TCR-expressing T cells among both pathogenic Th17 (pTh17) and Treg populations (72). These dual TCR T cells displayed features of clonal proliferation and reduced TCR repertoire diversity, particularly in synovial fluid samples, suggesting their involvement in ongoing autoimmune responses. Importantly, the authors found overlapping TCRβ CDR3 sequences between Th1, Th17, pTh17, and Treg subsets, indicating potential lineage interconversion or shared antigenic specificity. Dual TCR Tregs exhibited elevated expression of transcription factors such as Foxp3 and STAT5, yet paradoxically produced less IL-10 and more pro-inflammatory cytokines than their single TCR counterparts. These observations reinforce the notion that under inflammatory conditions, Tregs may acquire pathogenic features, thus contributing to chronic inflammation in AS.

**Figure 2:**
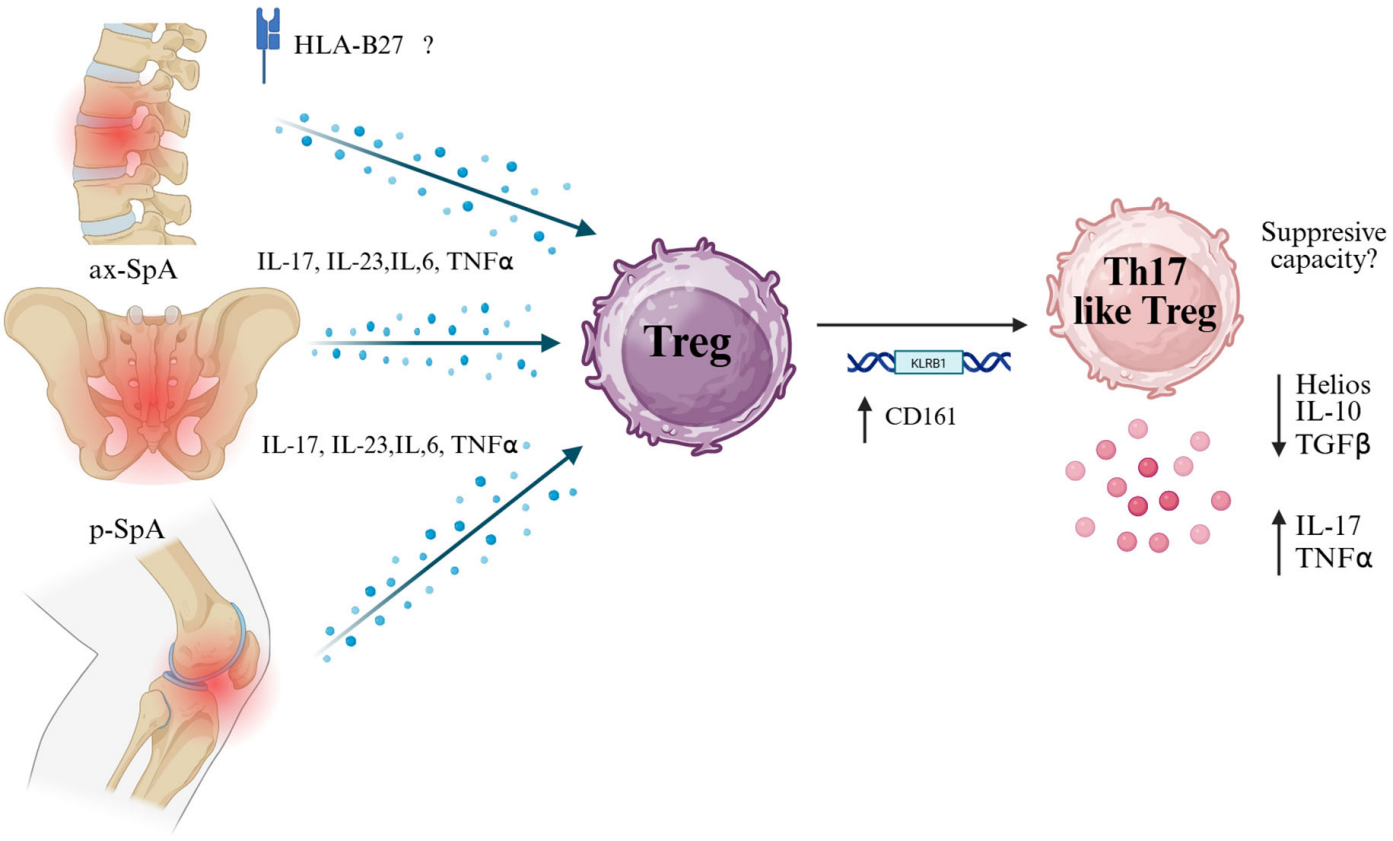
Pro-inflammatory cytokine milieu in spondyloarthritis may drive Treg plasticity toward a Th17-like phenotype. In both axial (ax-SpA) and peripheral (p-SpA) forms of spondyloarthritis, elevated levels of IL-17, IL-23, IL-6, and TNF-α — partly influenced by HLA-B27 — exposed regulatory T cells (Tregs) to inflammatory pressure. This environment may induce the expression of CD161 (encoded by KLRB1), a marker associated with Th17-like features. CD161+ Tregs have been reported to exhibit reduced suppressive capacity and gain pro-inflammatory functions, characterized by increased secretion of IL-17 and TNF-α, along with decreased expression of Helios, IL-10, and TGF-β. This transition suggests a pathogenic plasticity contributing to chronic inflammation in SpA. Created in BioRender. Romero, P (2025). https://BioRender.com/p29m2dc.

Accordingly, Treg dysfunction in SpA seems multifactorial; in homeostatic conditions, it involves a decrease in anti-inflammatory cytokines such as IL-10 and TGF-β (73). Nevertheless, the cues in SpA have not been studied in detail. The potential breakdown in regulatory capacity likely facilitates the chronic activation of autoreactive T cells and sustains the inflammatory loop in affected joints (**Figure 2**).

In the context of SpA, tissue-resident Tregs in the synovial fluid exhibit remarkable phenotypic and functional specialization. Recent studies have identified various subtypes among these Tregs, including those with a genetic profile associated with the Th17 lineage, characterized by *RORC* expression, IL-10 production, and LAG-3 presence, as well as CD161^+^ Tregs with low Helios expression and CD8^+^ Tregs with cytotoxic properties. These cells also show overexpression of genes related to interferon and IL-17 signatures, as well as members of the TNF receptor superfamily, reflecting adaptation to the inflamed joint environment. Additionally, significant clonal expansion has been observed, suggesting antigen-driven local responses. Functionally, these Tregs can suppress the secretion of pro-inflammatory cytokines such as IL-12/23 and TNF by monocytes via LAG-3, indicating a specialized regulatory mechanism in the joint environment. However, some Th17-like, Helios-negative, and IL-17A-producing Tregs are also abundant in synovial fluid and may play a dual role in local regulation and inflammation (20, 21). Although these cells were found in the synovial fluid, tissue-resident Tregs (resident to the bone, cartilage, or enthesis) have not been characterized yet.

While multiple studies have described phenotypic and transcriptomic alterations in Tregs, few have directly assessed their suppressive capacity or regulatory behavior *in vivo* or *ex vivo*. Detailed functional analyses are urgently needed to determine whether these altered cells actively contribute to disease progression or merely reflect bystander activation within the inflamed tissue. Moreover, the potential relationship between Treg instability and hallmark features of SpA—such as HLA-B27 positivity or aberrant osteoproliferation—remains poorly understood. Whether Treg dysregulation facilitates pathological bone remodeling, fails to control entheseal inflammation, or interacts with antigen presentation mechanisms influenced by HLA-B27 are open questions that warrant further investigation.

## 3 Treg plasticity in psoriasis

Psoriasis is a chronic, inflammatory, and immune-mediated skin disease characterized by hyperkeratosis that results in the formation of pruriginous plaques and systemic inflammation that significantly affects the patient’s quality of life (74). The underlying pathogenic mechanisms involve a complex interaction between the innate and adaptive immune systems (75). In this process, T cells interact with dendritic cells, macrophages, and keratinocytes; this immune cell network, along with a myriad of cytokines, initiates a chronic inflammatory response attributed to cytokines from Th1 and, particularly, Th17 phenotypes, the latter now considered the main effector in pathogenesis (76).

This disease primarily affects the skin, leading to excessive proliferation of keratinocytes, the cells responsible for forming the outermost layer of the epidermis. Under normal conditions, keratinocytes renew in a cycle of approximately 28 days; however, in psoriasis, this cycle accelerates drastically, shortening to just 3 or 4 days (77). This process is mainly mediated by T lymphocytes, which in psoriasis patients are inappropriately activated, triggering an inflammatory cascade. These activated CD4 T cells secrete pro-inflammatory cytokines such as TNF-α, IL-17, IL-23, and IL-22, promoting inflammation and stimulating rapid keratinocyte production (78).

Originally, Th1 cells were considered the main mediators of psoriasis due to the expression of IFN-γ in skin lesions and the peripheral blood of patients. However, more recent studies have identified a central role for Th17 cells in disease progression (79).This paradigm shift has directly influenced the development of targeted therapies: over the past decade, biological products directed at TNF-α, IL-23, and IL-17 have been developed and approved, drastically transforming the clinical management of the disease (78).

Antimicrobial peptides (AMPs), expressed in the epidermis, also play a key role in psoriasis pathogenesis, as they strongly induce IL-17 production and activate signaling cascades that stimulate the innate immune system. These peptides activate plasmacytoid dendritic cells, which release IFN-α, a cytokine that stimulates myeloid cells and promotes the maturation of dendritic cells. As a result, higher levels of cytokines such as IL-12, IL-23, and TNF-α are produced, which activate key T cell populations like Th1, Th17, and Th22, with Th17 being the most prominent in this disease (80).

Regarding immune regulation, it has been observed that the imbalance between regulatory T cells (Treg) and Th17 cells contributes to the exacerbation of skin inflammation, favoring the progression and maintenance of the disease. In inflammatory contexts such as psoriasis, Treg cells lose their suppressive capacity, enabling the overexpression of pro-inflammatory cytokines by Th17 cells. Notably, Treg cells producing IL-17 have been identified in psoriasis patients, suggesting a functional plasticity of these cells that, instead of suppressing, contribute to the inflammatory process (21, 81).

Furthermore, studies in pediatric psoriasis patients have shown a simultaneous increase in both Th17 and Treg cells in peripheral blood, with an elevated Th17/Treg ratio that positively correlates with the clinical severity of the disease. These findings indicate that Treg cell dysfunction may be present from the early stages of the disease and play a significant role in perpetuating the chronic inflammatory response (82).

**Figure 3:**
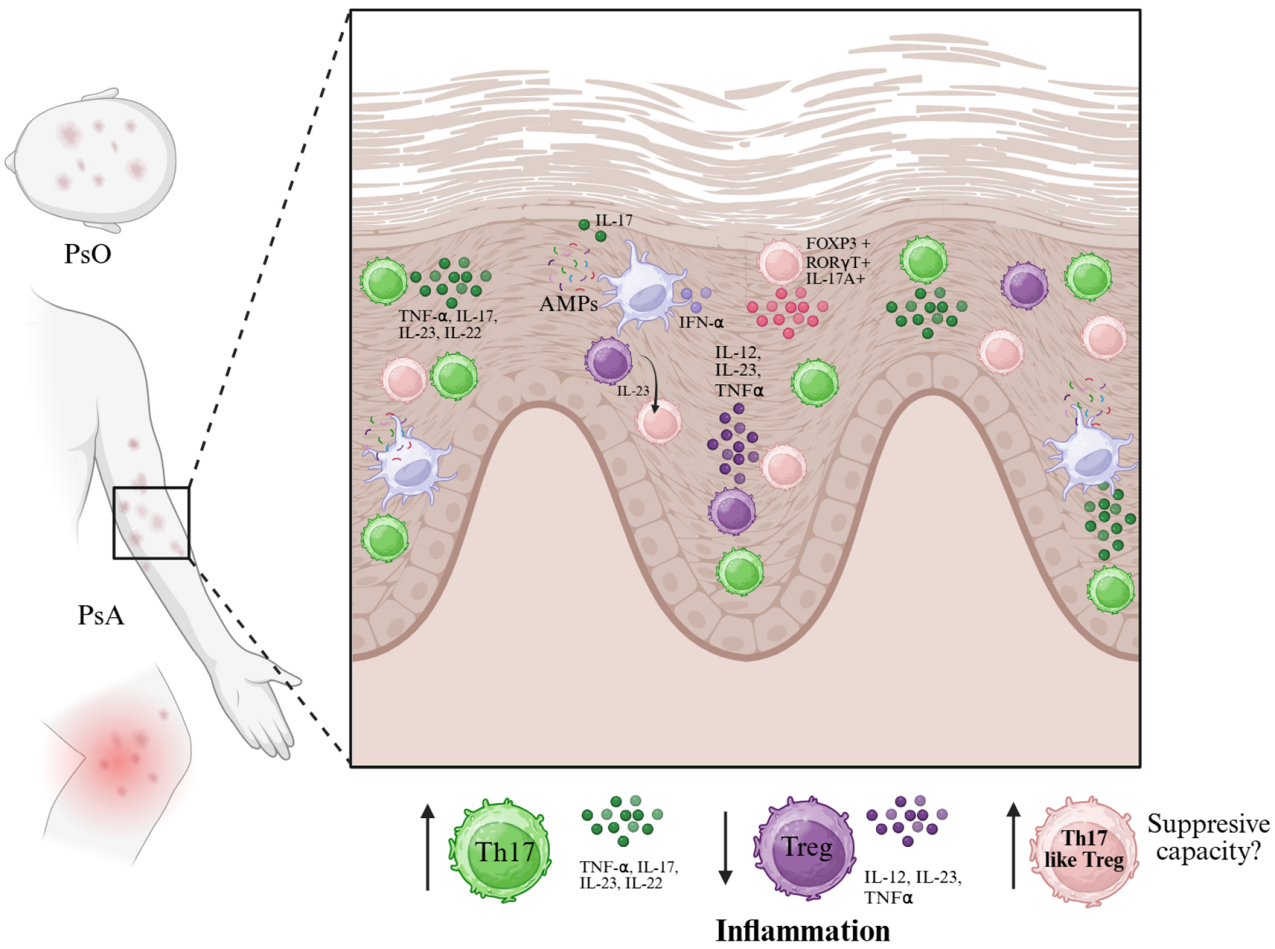
In psoriasis (PsO) and psoriatic arthritis (PsA), the skin microenvironment is enriched with pro-inflammatory cytokines such as IL-23, IL-12, IL-17, TNF-α, and IFN-α, largely produced by dendritic cells and other innate immune cells in response to antimicrobial peptides (AMPs). These signals promote the expansion of Th17 cells and may destabilize FoxP3⁺ regulatory T cells (Tregs), contributing to a shift toward a Th17-like phenotype. This process is characterized by the emergence of FoxP3^+^RORγt^+^IL-17A^+^ Tregs with reduced suppressive capacity. The accumulation of Th17 and Th17-like Treg cells, alongside diminished functional Tregs, leads to sustained inflammation in psoriatic lesions. The diagram illustrates the cytokine milieu and key cellular players involved in this dysregulated immune response at the epidermal level. Created in BioRender. Romero, P. (2025) https://BioRender.com/p29m2dc.

Over the last five years, multiple studies have demonstrated that Treg cells in psoriasis and psoriatic arthritis exhibit high plasticity, frequently losing their regulatory phenotype and producing inflammatory cytokines. This plasticity contributes to disease progression and represents a potential target for therapeutic intervention. This plasticity is observed both in human patients and in mouse models of psoriasiform dermatitis, where IL-23 drives the accumulation of Foxp3+RORγt+IL-17A+ Tregs in the skin (21, 81) (**Figure 3**).

Treg cells from psoriasis patients show an increased tendency to differentiate into IL-17A-producing cells, often expressing the Th17-associated transcription factor RORγt and sometimes losing Foxp3 expression in a mechanism mediated by STAT3 (83).

Moreover, in lesional skin and inflamed joints, Tregs with intermediate levels of FoxP3 expression can be the main source of IL-17A and express markers such as CD161 and RORγt (83). As well as circulating cells, skin-resident Tregs may lose their typical suppressive function and acquire a pro-inflammatory profile, characterized by IL-17A production. This shift contributes to the immune imbalance that underlies the disease, altering the local immune regulation of the balance between Tregs and tissue-resident memory T cells (TRMs). From a therapeutic perspective, targeting the IL-17A and IL-23 pathways has proven effective in modulating the Treg/TRM ratio. Notably, IL-23 inhibition appears to offer longer-lasting benefits by restoring Treg regulatory function (84).

In psoriasis, IL-23 emerged as a central cytokine in modulating Treg plasticity. Kannan et al. demonstrated in a murine model that IL-23 promotes the generation of CD4^+^FoxP3^+^RORγt^+^IL-17A^+^ cells, both *in vivo* and *in vitro*, indicating that IL-23 exposure destabilizes the regulatory program and facilitates conversion into IL-17-producing cells. Pharmacological inhibition of RORγt reversed this phenotype, suggesting that this axis could be therapeutically targetable (85).

In psoriasis (PsO) and psoriatic arthritis (PsA), the skin microenvironment is enriched with pro-inflammatory cytokines such as IL-23, IL-12, IL-17, TNF-α, and IFN-α, largely produced by dendritic cells and other innate immune cells in response to antimicrobial peptides (AMPs). These signals promote the expansion of Th17 cells and may destabilize FoxP3^+^ regulatory T cells (Tregs), contributing to a shift toward a Th17-like phenotype. This process is characterized by the emergence of FoxP3^+^RORγt^+^IL-17A^+^ Tregs with reduced suppressive capacity. The accumulation of Th17 and Th17-like Treg cells, alongside diminished functional Tregs, leads to sustained inflammation in psoriatic lesions. The diagram illustrates the cytokine milieu and key cellular players involved in this dysregulated immune response at the epidermal level. Created in BioRender. Romero, P (2025). https://BioRender.com/p29m2dc.

## 4 Concluding remarks

Despite growing interest in effector T cell subsets in SpA and PsO, the study of Treg plasticity has remained comparatively underexplored. Much of the literature focuses on characterizing the inflammatory milieu, with less attention given to regulatory mechanisms that might counterbalance such responses. However, recent work has identified key pathways and factors that modulate the Treg–Th17 axis. For example, in an imiquimod-induced mouse model of psoriasis, melatonin was shown to preserve Treg stability and dampen Th17-mediated inflammation, suggesting novel avenues for therapeutic intervention (86).

Further supporting this concept, a study using a murine model of spondyloarthritis demonstrated that treatment with the peptide VnP-16—either alone or in combination with the COX-2 inhibitor celecoxib—significantly reduced clinical disease scores. This improvement was accompanied by reduced levels of IL-1β, IL-6, TNF-α, and IL-17 and restoration of the Th17/Treg balance (87). Notably, VnP-16 promoted polarization toward a Treg phenotype, as evidenced by increased FoxP3 expression and suppression of STAT3 phosphorylation, a pathway critical for Th17 differentiation.

In a complementary model using HLA-B27/hβ2m transgenic rats, an excess of effector Th17 cells was observed alongside Tregs expressing elevated levels of ICOS. Although ICOS is generally associated with enhanced suppressive function and IL-10 production, these Tregs paradoxically exhibited reduced IL-10 and increased IL-17 expression. Blocking ICOS signaling during Treg differentiation *in vitro* restored the IL-10/IL-17 balance, stressing the role of aberrant ICOS signaling in driving Treg plasticity in SpA (88).

Additional mechanisms have also been implicated in Treg plasticity in psoriatic and SpA contexts. These include aberrant co-stimulatory signals such as ICOS (88), low Helios expression (69), epigenetic instability at the FoxP3 locus (89) and local metabolic or hypoxic conditions that regulate FoxP3 stability (34).

Understanding the molecular pathways that regulate Treg stability and plasticity is essential for developing new therapeutic strategies. Future interventions to restore Treg function or prevent their pathogenic conversion may offer novel approaches to control chronic inflammation in SpA and PsO. These can include cytokines or metabolic pathways, such as fatty acid oxidation (FAO), which contribute to Treg cells’ suppressive function and stability. Targeting these pathways may offer promising therapeutic strategies in chronic inflammatory disease management (52).

Although significant differences exist between human disease and murine models, animal studies remain crucial for elucidating the dynamics of Treg cell origin, fate, and plasticity. For instance, Foxp3 fate-mapping models allow for the identification of cells that once expressed Foxp3 but have since lost it—so-called “ex-Tregs”—offering direct insights into the loss of regulatory identity and potential acquisition of effector functions (90). Integrating such technologies will be essential for understanding the molecular mechanisms of Treg plasticity and for establishing the rational design and optimization of Treg-based immunotherapies.

Collectively, these findings highlight that Tregs are not a fixed population, but rather a plastic and dynamic cell type whose behavior is profoundly shaped by the local microenvironment. Understanding the molecular cues that regulate their identity and function is critical to unlocking their therapeutic potential in autoinflammatory diseases. Future research should focus on dissecting the context-dependent signals that drive Treg instability, the tissue-specific factors that influence their fate, and the long-term consequences of Treg plasticity on disease progression and treatment resistance.

## References

[1] Sakaguchi S Sakaguchi N Asano M Itoh M Toda M. Immunologic self-tolerance maintained by activated T cells expressing IL-2 receptor alpha-chains (CD25). Breakdown of a single mechanism of self-tolerance causes various autoimmune diseases. J Immunol. (1995) 155:1151–64. doi: 10.4049/jimmunol.155.3.1151 7636184

[2] Takahashi T Kuniyasu Y Toda M Sakaguchi N Itoh M Iwata M. Immunologic self-tolerance maintained by CD25+CD4+ naturally anergic and suppressive T cells: Induction of autoimmune disease by breaking their anergic/suppressive state. Int Immunol. (1998) 10:1969–80. doi: 10.1093/intimm/10.12.1969, PMID: 9885918

[3] Tarr TK Jameson SC Hogquist KA. Positive and negative selection of T cells. Annu Rev Immunol. (2003) 21:139–76. doi: 10.1146/annurev.immunol.21.120601.141107, PMID: 12414722

[4] González Parias JL Duque Giraldo VE Velásquez-Lopera MM. FoxP3: Master regulator of the generation and function of natural regulatory T cells. Inmunología. (2010) 29:74–84. doi: 10.1016/S0213-9626(10)70013-5

[5] Hori S Nomura T Sakaguchi S. Control of regulatory T cell development by the transcription factor Foxp3. J Immunol. (2017) 198:981–5. doi: 10.1126/science.1079490, PMID: 28115586

[6] Ohkura N Hamaguchi M Morikawa H Sugimura K Tanaka A Ito Y. T cell receptor stimulation-induced epigenetic changes and Foxp3 expression are independent and complementary events required for Treg cell development. Immunity. (2012) 37:785–99. doi: 10.1016/j.immuni.2012.09.010, PMID: 23123060

[7] Kanamori M Nakatsukasa H Okada M Lu Q Yoshimura A. Induced regulatory T cells: Their development, stability, and applications. Trends Immunol. (2016) 37:803–11. doi: 10.1016/j.it.2016.08.012, PMID: 27623114

[8] Shevach EM Thornton AM. tTregs, pTregs, and iTregs: similarities and differences. Immunol Rev. (2014) 259:88–102. doi: 10.1111/imr.12160, PMID: 24712461 PMC3982187

[9] Hui Z Zhang J Zheng Y Yang L Yu W An Y. Single-cell sequencing reveals the transcriptome and TCR characteristics of pTregs and *in vitro* expanded iTregs. Front Immunol. (2021) 12:619932. doi: 10.3389/fimmu.2021.619932, PMID: 33868236 PMC8044526

[10] Shevach EM. Mechanisms of foxp3+ T regulatory cell-mediated suppression. Immunity. (2009) 30:636–45. doi: 10.1016/j.immuni.2009.04.010, PMID: 19464986

[11] Walker LS Sansom DM. The emerging role of CTLA4 as a cell-extrinsic regulator of T cell responses. Nat Rev Immunol. (2011) 11:852–63. doi: 10.1038/nri3108, PMID: 22116087

[12] Cai J Wang D Zhang G Guo X. The role of PD-1/PD-L1 axis in treg development and function: Implications for cancer immunotherapy. Onco Targets Ther. (2019) 12:8437–45. doi: 10.2147/OTT.S221340, PMID: 31686860 PMC6800566

[13] Wei X Zhang J Gu Q Huang M Zhang W Guo J. Reciprocal expression of IL-35 and IL-10 defines two distinct effector Treg subsets that are required for maintenance of immune tolerance. Cell Rep. (2017) 21:1853–65. doi: 10.1016/j.celrep.2017.10.090, PMID: 29141218

[14] Francisco LM Salinas VH Brown KE Vanguri VK Freeman GJ Kuchroo VK. PD-L1 regulates the development, maintenance, and function of induced regulatory T cells. J Exp Med. (2009) 206:3015–29. doi: 10.1084/jem.20090847, PMID: 20008522 PMC2806460

[15] Zhao H Liao X Kang Y. Tregs: Where we are and what comes next? Front Immunol. (2017) 8:1578. doi: 10.3389/fimmu.2017.01578, PMID: 29225597 PMC5705554

[16] Lykhopiy V Malviya V Humblet-Baron S Schlenner SM. IL-2 immunotherapy for targeting regulatory T cells in autoimmunity. Genes Immun. (2023) 24:248–62. doi: 10.1038/s41435-023-00221-y, PMID: 37741949 PMC10575774

[17] Zou T Caton AJ Koretzky GA Kambayashi T. Dendritic cells induce regulatory T cell proliferation through antigen-dependent and -independent interactions. J Immunol. (2010) 185:2790–9. doi: 10.4049/jimmunol.0903740, PMID: 20686126

[18] Contreras-Castillo E García-Rasilla VY García-Patiño MG Licona-Limón P. Stability and plasticity of regulatory T cells in health and disease. J Leukoc Biol. (2024) 116:33–53. doi: 10.1093/JLEUKO/QIAE049, PMID: 38428948

[19] Köhler A Geiselhöringer AL Kolland D Kreft L Wichmann N Hils M. The atypical IκB family member Bcl3 determines differentiation and fate of intestinal RORγt+ regulatory T cell subsets. Mucosal Immunol. (2024) 17:673–91. doi: 10.1016/J.MUCIMM.2024.04.002, PMID: 38663461

[20] Simone D Penkava F Ridley A Sansom S Al-Mossawi H Bowness P. Single cell analysis of spondyloarthritis regulatory T cells identifies distinct 2 synovial gene expression patterns and clonal fates. Commun Biol. (2021) 4 (1):1395. doi: 10.1038/s42003-021-02931-3, PMID: 34907325 PMC8671562

[21] Pouw J Nordkamp MO Van Kempen T Concepcion A Van Laar J Van Wijk F. Regulatory T cells in psoriatic arthritis: an IL-17A-producing, Foxp3intCD161 + RORγt + ICOS + phenotype, that associates with the presence of ADAMTSL5 autoantibodies. Sci Rep. (2022) 12(1):20675. doi: 10.1038/s41598-022-24924-w, PMID: 36450783 PMC9712434

[22] Pietraforte I Frasca L. Autoreactive T-cells in psoriasis: are they spoiled tregs and can therapies restore their functions? Int J Mol Sci. (2023) 24(5):4348. doi: 10.3390/IJMS24054348, PMID: 36901778 PMC10002349

[23] Stock P Akbari O Berry G Freeman GJ Dekruyff RH Umetsu DT. Induction of T helper type 1-like regulatory cells that express Foxp3 and protect against airway hyper-reactivity. Nat Immunol. (2004) 5:1149–56. doi: 10.1038/ni1122, PMID: 15448689

[24] Warunek J Jin RM Blair SJ Garis M Marzullo B Wohlfert EA. Tbet expression by regulatory T cells is needed to protect against Th1-mediated immunopathology during Toxoplasma infection in mice. Immunohorizons. (2021) 5:931–43. doi: 10.4049/immunohorizons.2100080, PMID: 34893511

[25] Miyao T Floess S Setoguchi R Luche H Fehling HJ Waldmann H. Plasticity of Foxp3(+) T cells reflects promiscuous Foxp3 expression in conventional T cells but not reprogramming of regulatory T cells. Immunity. (2012) 36:262–75. doi: 10.1016/j.immuni.2011.12.012, PMID: 22326580

[26] Hori S. Lineage stability and phenotypic plasticity of Foxp3^+^ regulatory T cells. Immunol Rev. (2014) 259 (1):159–172. doi: 10.1111/imr.12175, PMID: 24712465

[27] Ferreira RC Simons HZ Thompson WS Rainbow DB Yang X Cutler AJ. Cells with Treg-specific FoxP3 demethylation but low CD25 are prevalent in autoimmunity. J Autoimmun. (2017) 84:75–86. doi: 10.1016/j.jaut.2017.07.009, PMID: 28747257 PMC5656572

[28] Polansky JK Kretschmer K Freyer J Floess S Garbe A Baron U. DNA methylation controls Foxp3 gene expression. Eur J Immunol. (2008) 38:1654–63. doi: 10.1002/eji.200838105, PMID: 18493985

[29] Zhu L Liu Z Cui Q Guan G Hui R Wang X. Epigenetic modification of CD4+ T cells into Tregs by 5-azacytidine as cellular therapeutic for atherosclerosis treatment. Cell Death Dis. (2024) 15(9):689. doi: 10.1038/S41419-024-07086-7, PMID: 39304654 PMC11415506

[30] Göschl L Scheinecker C Bonelli M. Treg cells in autoimmunity: from identification to Treg-based therapies. Semin Immunopathol. (2019) 41:301–14. doi: 10.1007/s00281-019-00741-8, PMID: 30953162

[31] Shan J Feng L Sun G Chen P Zhou Y Xia M. Interplay between mTOR and STAT5 signaling modulates the balance between regulatory and effective T cells. Immunobiology. (2015) 220:510–7. doi: 10.1016/j.imbio.2014.10.020, PMID: 25468562

[32] Arteaga-Cruz S Cortés-Hernández A Alvarez-Salazar EK Rosas-Cortina K Aguilera-Sandoval C Morales-Buenrostro LE. Highly purified and functionally stable *in vitro* expanded allospecific Tr1 cells expressing immunosuppressive graft-homing receptors as new candidates for cell therapy in solid organ transplantation. Front Immunol. (2023) 14:1062456. doi: 10.3389/FIMMU.2023.1062456, PMID: 36911743 PMC9998667

[33] Qu Y Zhang B Zhao L Liu G Ma H Rao E. The effect of immunosuppressive drug rapamycin on regulatory CD4+CD25+Foxp3+T cells in mice. Transpl Immunol. (2007) 17:153–61. doi: 10.1016/j.trim.2007.01.002, PMID: 17331841

[34] Gu H Wang Z Xie X Chen H Ouyang J Wu R. HIF-1α induced by hypoxic condition regulates Treg/Th17 axis polarization in chronic immune thrombocytopenia. Int Immunopharmacol. (2024) 131:111810. doi: 10.1016/J.INTIMP.2024.111810, PMID: 38492341

[35] Koch MA Tucker-Heard G Perdue NR Killebrew JR Urdahl KB Campbell DJ. The transcription factor T-bet controls regulatory T cell homeostasis and function during type 1 inflammation. Nat Immunol. (2009) 10:595–602. doi: 10.1038/ni.1731, PMID: 19412181 PMC2712126

[36] Kitz A Dominguez-Villar M. Molecular mechanisms underlying Th1-like Treg generation and function. Cell Mol Life Sci. (2017) 74:4059–75. doi: 10.1007/S00018-017-2569-Y, PMID: 28624966 PMC7079789

[37] Halim L Romano M McGregor R Correa I Pavlidis P Grageda N. An atlas of human regulatory T helper-like cells reveals features of th2-like tregs that support a tumorigenic environment. Cell Rep. (2017) 20:757–70. doi: 10.1016/j.celrep.2017.06.079, PMID: 28723576 PMC5529316

[38] Nyirenda MH Sanvito L Darlington PJ O’Brien K Zhang G-X Constantinescu CS. TLR2 stimulation drives human naive and effector regulatory T cells into a th17-like phenotype with reduced suppressive function. J Immunol. (2011) 187:2278–90. doi: 10.4049/jimmunol.1003715, PMID: 21775683

[39] Lee YK Mukasa R Hatton RD Weaver CT. Developmental plasticity of Th17 and Treg cells. Curr Opin Immunol. (2009) 21:274–80. doi: 10.1016/j.coi.2009.05.021, PMID: 19524429

[40] Chung Y Tanaka S Chu F Nurieva RI Martinez GJ Rawal S. Follicular regulatory T cells expressing Foxp3 and Bcl-6 suppress germinal center reactions. Nat Med. (2011) 17:983–8. doi: 10.1038/nm.2426, PMID: 21785430 PMC3151340

[41] Powell MD Read KA Sreekumar BK Oestreich KJ. Ikaros zinc finger transcription factors: Regulators of cytokine signaling pathways and CD4+ T helper cell differentiation. Front Immunol. (2019) 10:1299/BIBTEX. doi: 10.3389/FIMMU.2019.01299/BIBTEX, PMID: 31244845 PMC6563078

[42] Thornton AM Shevach EM. Helios: still behind the clouds. Immunology. (2019) 158:161–70. doi: 10.1111/imm.13115, PMID: 31517385 PMC6797934

[43] Thornton AM Lu J Korty PE Kim YC Martens C Sun PD. Helios+ and Helios- Treg subpopulations are phenotypically and functionally distinct and express dissimilar TCR repertoires. Eur J Immunol. (2019) 49:398–412. doi: 10.1002/eji.201847935, PMID: 30620397 PMC6402968

[44] Getnet D Grosso JF Goldberg MV Harris TJ Yen HR Bruno TC. A role for the transcription factor Helios in human CD4(+)CD25(+) regulatory T cells. Mol Immunol. (2010) 47:1595–600. doi: 10.1016/j.molimm.2010.02.001, PMID: 20226531 PMC3060613

[45] Ayyoub M Raffin C Valmori D. Comment on “Helios + and helios – cells coexist within the natural foxP3 + T regulatory cell subset in humans. J Immunol. (2013) 190:4439. doi: 10.4049/jimmunol.1390018, PMID: 23606718

[46] Takatori H Kawashima H Matsuki A Meguro K Tanaka S Iwamoto T. Helios enhances treg cell function in cooperation with foxP3. Arthritis Rheumatol. (2015) 67:1491–502. doi: 10.1002/art.39091, PMID: 25733061

[47] Baine I Basu S Ames R Sellers RS Macian F. Helios induces epigenetic silencing of il2 gene expression in regulatory T cells. J Immunol. (2013) 190:1008–16. doi: 10.4049/JIMMUNOL.1200792, PMID: 23275607 PMC3558938

[48] Sebastian M Lopez-Ocasio M Metidji A Rieder SA Shevach EM Thornton AM. Helios controls a limited subset of regulatory T cell functions. J Immunol. (2016) 196:144–55. doi: 10.4049/JIMMUNOL.1501704, PMID: 26582951 PMC4685018

[49] Thornton AM Kilaru G Burr P Rieder S Muljo SA Shevach EM. Helios expression defines two distinct populations of Foxp3+ regulatory T cells. J Immunol. (2016) 196:125. doi: 10.4049/JIMMUNOL.196.SUPP.125.6

[50] Hatzioannou A Boumpas A Papadopoulou M Papafragkos I Varveri A Alissafi T. Regulatory T cells in autoimmunity and cancer: A duplicitous lifestyle. Front Immunol. (2021) 12:731947. doi: 10.3389/FIMMU.2021.731947, PMID: 34539668 PMC8446642

[51] Freuchet A Roy P Armstrong SS Oliaeimotlagh M Kumar S Orecchioni M. Identification of human exTreg cells as CD16+CD56+ cytotoxic CD4+ T cells. Nat Immunol. (2023) 24:1748–61. doi: 10.1038/S41590-023-01589-9;SUBJMETA=1619,1898,250,38,554,631;KWRD=AUTOIMMUNITY,CD4-POSITIVE+T+CELLS, PMID: 37563308 PMC11022744

[52] Shi H Chi H. Metabolic control of Treg cell stability, plasticity, and tissue-specific heterogeneity. Front Immunol. (2019) 10:2716. doi: 10.3389/fimmu.2019.02716, PMID: 31921097 PMC6917616

[53] Lyu Q. Mechanism of Treg conversion to exTreg in atherosclerosis. J Immunol. (2024) 212(8):1956–67. doi: 10.4049/JIMMUNOL.212.SUPP.0223.5677

[54] Fletcher J Lonergan R Costelloe L Kinsella K Moran B O’Farrelly C. CD39+Foxp3+ Regulatory T cells suppress pathogenic th17 cells and are impaired in multiple sclerosis1. J Immunol. (2009) 183:7602–10. doi: 10.4049/jimmunol.0901881, PMID: 19917691

[55] Wang T Sun X Zhao J Zhang J Zhu H Li C. Regulatory T cells in rheumatoid arthritis showed increased plasticity toward Th17 but retained suppressive function in peripheral blood. Ann Rheum Dis. (2014) 74:1293–301. doi: 10.1136/annrheumdis-2013-204228, PMID: 24521740

[56] Miyabe C Miyabe Y Strle K Kim ND Stone J Luster A. An expanded population of pathogenic regulatory T cells in giant cell arteritis is abrogated by IL-6 blockade therapy. Ann Rheum Dis. (2016) 76:898–905. doi: 10.1136/annrheumdis-2016-210070, PMID: 27927642 PMC5744591

[57] Chen J Ye H Xiao W Mao Y Ai S Chen R. Increased dysfunctional and plastic regulatory T cells in idiopathic orbital inflammation. Front Immunol. (2021) 12:634847. doi: 10.3389/fimmu.2021.634847, PMID: 34012433 PMC8126653

[58] Zhou J Felix FA Jiang Y Li D Kim MC Jang D. Altered characteristics of regulatory T cells in target tissues of Sjögren’s syndrome in murine models. Mol Immunol. (2024) 174:47–56. doi: 10.1016/j.molimm.2024.08.003, PMID: 39197397 PMC11500054

[59] Sieper J Poddubnyy D. Axial spondyloarthritis. Lancet. (2017) 390:73–84. doi: 10.1016/S0140-6736(16)31591-4, PMID: 28110981

[60] McMichael A Bowness P. HLA-B27: natural function and pathogenic role in spondyloarthritis. Arthritis Res. (2002) 4 Suppl 3:S153–8. doi: 10.1186/ar571, PMID: 12110134 PMC3240147

[61] Rudwaleit M Landewé R Sieper J. Ankylosing spondylitis and axial spondyloarthritis. N Engl J Med. (2016) 375:1302–3. doi: 10.1056/NEJMc1609622, PMID: 27682052

[62] Romero-López JP Elewaut D Pacheco-Tena C Burgos-Vargas R. Inflammatory foot involvement in spondyloarthritis: from tarsitis to ankylosing tarsitis. Front Med (Lausanne). (2021) 8:730273. doi: 10.3389/fmed.2021.730273, PMID: 34692724 PMC8531414

[63] Danve A Deodhar A. Treatment of axial spondyloarthritis: an update. Nat Rev Rheumatol. (2022) 18:205–16. doi: 10.1038/s41584-022-00761-z, PMID: 35273385

[64] Deodhar A Mease P Rahman P Navarro-Compán V Marzo-Ortega H Hunter T. Ixekizumab improves patient-reported outcomes in non-radiographic axial spondyloarthritis: results from the coast-X trial. Rheumatol Ther. (2021) 8:135–50. doi: 10.1007/s40744-020-00254-z, PMID: 33284423 PMC7991024

[65] Schett G Baraliakos X Van den Bosch F Deodhar A Østergaard M Das GA. Secukinumab efficacy on enthesitis in patients with ankylosing spondylitis: pooled analysis of four pivotal phase III studies. J Rheumatol. (2021) 48:1251–8. doi: 10.3899/jrheum.201111, PMID: 33722947

[66] McInnes IB Szekanecz Z McGonagle D Maksymowych WP Pfeil A Lippe R. A review of JAK-STAT signalling in the pathogenesis of spondyloarthritis and the role of JAK inhibition. Rheumatol (Oxford). (2022) 61:1783–94. doi: 10.1093/rheumatology/keab740, PMID: 34668515 PMC9071532

[67] Lai NL Zhang SX Wang J Zhang JQ Wang CH Gao C. The proportion of regulatory T cells in patients with ankylosing spondylitis: A meta-analysis. J Immunol Res. (2019) 2019:1058738. doi: 10.1155/2019/1058738, PMID: 31772947 PMC6854227

[68] McTaggart T Lim JX Smith KJ Heaney B McDonald D Hulme G. Deep phenotyping of T regulatory cells in psoriatic arthritis highlights targetable mechanisms of disease. J Biol Chem. (2025) 301:108059. doi: 10.1016/j.jbc.2024.108059, PMID: 39662827 PMC11750473

[69] Simone D Penkava F Ridley A Sansom S Al-Mossawi MH Bowness P. Single cell analysis of spondyloarthritis regulatory T cells identifies distinct synovial gene expression patterns and clonal fates. Commun Biol. (2021) 4(1):1395. doi: 10.1038/s42003-021-02931-3, PMID: 34907325 PMC8671562

[70] Howie D Izcue A Lombardi G Duurland CL Brown CC O’shaughnessy RFL. cD161 + Tconv and cD161 + Treg share a Transcriptional and Functional Phenotype despite limited Overlap in Tcrβ repertoire. Immunol. (2017) 8:103. doi: 10.3389/fimmu.2017.00103, PMID: 28321213 PMC5337494

[71] Mebrek ML Abaab T Lemeiter D Breckler M Hervé R Petit M. Impairment of regulatory T cell stability in axial spondyloarthritis: role of EZH2 and pSTAT5. Front Immunol. (2024) 15:1484321. doi: 10.3389/FIMMU.2024.1484321, PMID: 39569199 PMC11576896

[72] Xu Y Peng Q Ma Q Yao X. scRNA + TCR-seq revealed the dual TCR pTh17 and Treg T cells involvement in autoimmune response in ankylosing spondylitis. Int Immunopharmacol. (2024) 135:112279. doi: 10.1016/j.intimp.2024.112279, PMID: 38796963

[73] Zong Y Deng K Chong WP. Regulation of Treg cells by cytokine signaling and co-stimulatory molecules. Front Immunol. (2024) 15:1387975/XML/NLM. doi: 10.3389/FIMMU.2024.1387975/XML/NLM, PMID: 38807592 PMC11131382

[74] Boehncke WH Schön MP. Psoriasis. Lancet. (2015) 386:983–94. doi: 10.1016/S0140-6736(14)61909-7, PMID: 26025581

[75] Man AM Orăsan MS Hoteiuc OA Olănescu-Vaida-Voevod MC Mocan T. Inflammation and psoriasis: A comprehensive review. Int J Mol Sci. (2023) 24:16095. doi: 10.3390/IJMS242216095, PMID: 38003284 PMC10671208

[76] Francis L Capon F Smith CH Haniffa M. Inflammatory memory in psoriasis: from remission to recurrence. J Allergy Clin Immunol. (2024) 154:42–50. doi: 10.1016/j.jaci.2023.07.017, PMID: 38761994

[77] Kamata M Tada Y. Crosstalk: keratinocytes and immune cells in psoriasis. Front Immunol. (2023) 14:1286344. doi: 10.3389/fimmu.2023.1286344, PMID: 38022549 PMC10665858

[78] Mylle S Grine L Speeckaert R Lambert JLW van Geel N. Targeting the IL-23/IL-17 pathway in psoriasis: the search for the good, the bad and the ugly. Am J Clin Dermatol. (2018) 19:625–37. doi: 10.1007/s40257-018-0366-5, PMID: 30003497

[79] Li B Huang L Lv P Li X Liu G Chen Y. The role of Th17 cells in psoriasis. Immunol Res. (2020) 68:296–309. doi: 10.1007/s12026-020-09149-1, PMID: 32827097

[80] Alecu M Coman G Muşetescu A Coman O. Antimicrobial peptides as an argument for the involvement of innate immunity in psoriasis (Review). Exp Ther Med. (2020) 20:1–1. doi: 10.3892/ETM.2020.9322, PMID: 33101482 PMC7579775

[81] Bovenschen HJ van de Kerkhof PC van Erp PE Woestenenk R Joosten I Koenen HJ. Foxp3+ regulatory T cells of psoriasis patients easily differentiate into IL-17A-producing cells and are found in lesional skin. J Invest Dermatol. (2011) 131:1853–60. doi: 10.1038/jid.2011.139, PMID: 21654831

[82] Zhang L Li Y Yang X Wei J Zhou S Zhao Z. Characterization of th17 and foxP3+ Treg cells in paediatric psoriasis patients. Scand J Immunol. (2016) 83:174–80. doi: 10.1111/SJI.12404, PMID: 26679087

[83] Yang L Li B Dang E Jin L Fan X Wang G. Impaired function of regulatory T cells in patients with psoriasis is mediated by phosphorylation of STAT3. J Dermatol Sci. (2016) 81:85–92. doi: 10.1016/J.JDERMSCI.2015.11.007, PMID: 26627723

[84] Lobão B Lourenço D Giga A Mendes-Bastos P. From PsO to PsA: the role of TRM and Tregs in psoriatic disease, a systematic review of the literature. Front Med (Lausanne). (2024) 11:1346757/XML/NLM. doi: 10.3389/FMED.2024.1346757/XML/NLM, PMID: 38405187 PMC10884248

[85] Kannan AK Su Z Gauvin DM Paulsboe SE Duggan R Lasko LM. IL-23 induces regulatory T cell plasticity with implications for inflammatory skin diseases. Sci Rep. (2019) 9:1–8. doi: 10.1038/s41598-019-53240-z, PMID: 31776355 PMC6881359

[86] Shen Z Jiang J Zhou X Tan Q Yan S Wu X. Melatonin attenuates imiquimod-induced psoriasis-like inflammation and restores the th17/treg immune balance. Inflammation. (2024) 47:2027–40. doi: 10.1007/s10753-024-02023-4, PMID: 38653920

[87] Min HK Choi JW Lee SY Ram Lee A Min BM Cho M. Vitronectin-derived bioactive peptide prevents spondyloarthritis by modulating Th17/Treg imbalance in mice with curdlan-induced spondyloarthritis. PloS One. (2022) 17:e0262183. doi: 10.1371/JOURNAL.PONE.0262183, PMID: 34986165 PMC8730421

[88] Araujo LM Fert I Jouhault Q Labroquère K Andrieu M Chiocchia G. Increased production of interleukin-17 over interleukin-10 by treg cells implicates inducible costimulator molecule in experimental spondyloarthritis. Arthritis Rheumatol. (2014) 66:2412–22. doi: 10.1002/ART.38737, PMID: 24909668

[89] Zhang Z Zhang W Guo J Gu Q Zhu X Zhou X. Activation and functional specialization of regulatory T cells lead to the generation of foxp3 instability. J Immunol. (2017) 198:2612–25. doi: 10.4049/JIMMUNOL.1601409/-/DCSUPPLEMENTAL, PMID: 28228556

[90] Simonetti M Yilmazer A Kretschmer K. Genetic tools for analyzing foxp3+ Treg cells: fluorochrome-based transcriptional reporters and genetic fate-mapping. Methods Mol Biol. (2023) 2559:95–114. doi: 10.1007/978-1-0716-2647-4_8/FIGURES/8, PMID: 36180629

